# Supplementary material for: Clostridium tetani bacteraemia in the plague area in France: Two cases
Source: Curr Res Microb Sci. 2025 Jan 9;8:100339. doi: 10.1016/j.crmicr.2025.100339 (PMC11786803; doi:10.1016/j.crmicr.2025.100339)
Supplement: Supplementary file 1 [file mmc1.pdf]

Supplementary figure 1: Krona Pie chart representing the taxonomic sequence classification by Kraken2 on galaxy Europe (Galaxy, https://usegalaxy.eu/)

I7 – Run Q7451

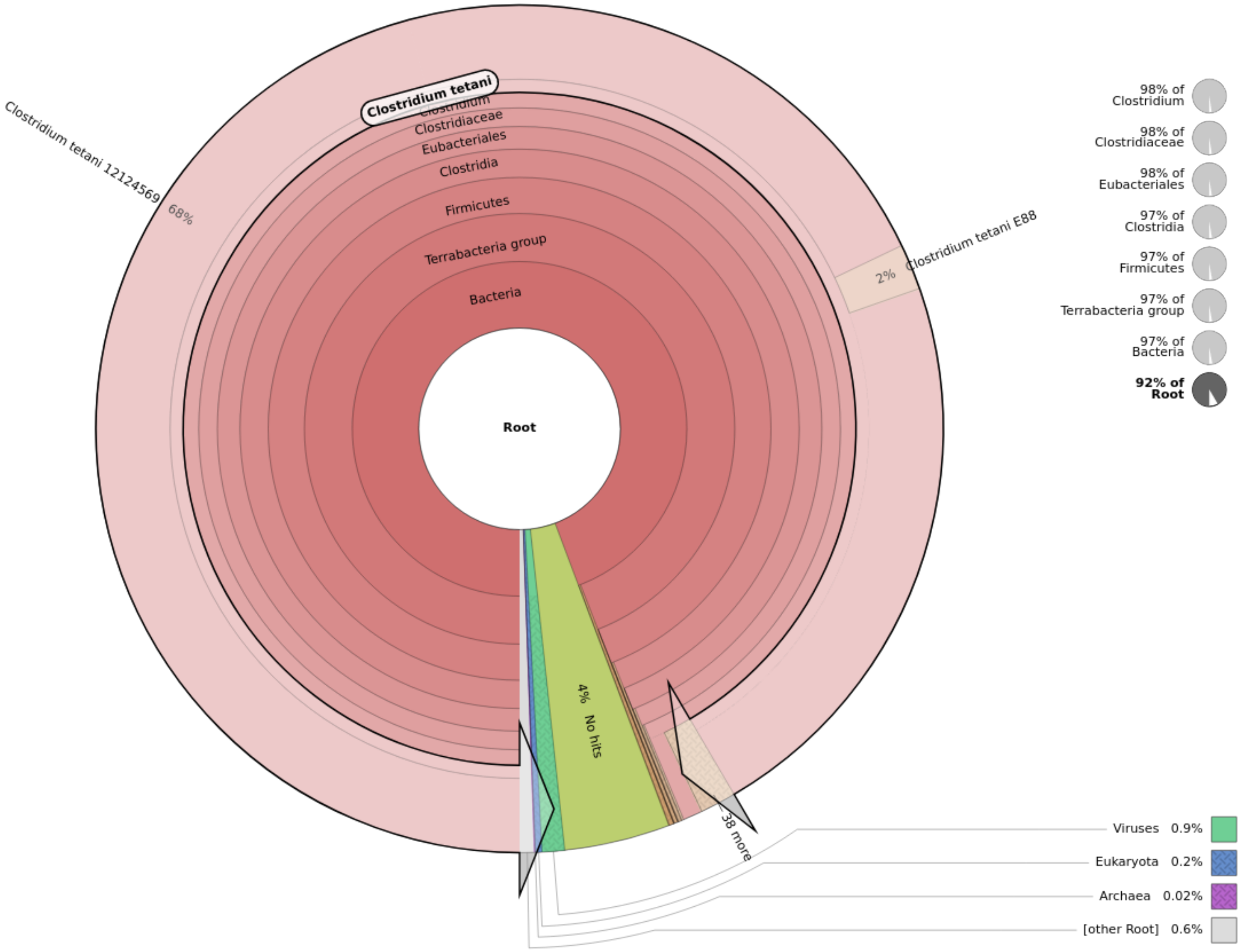

I44- Run 7452b

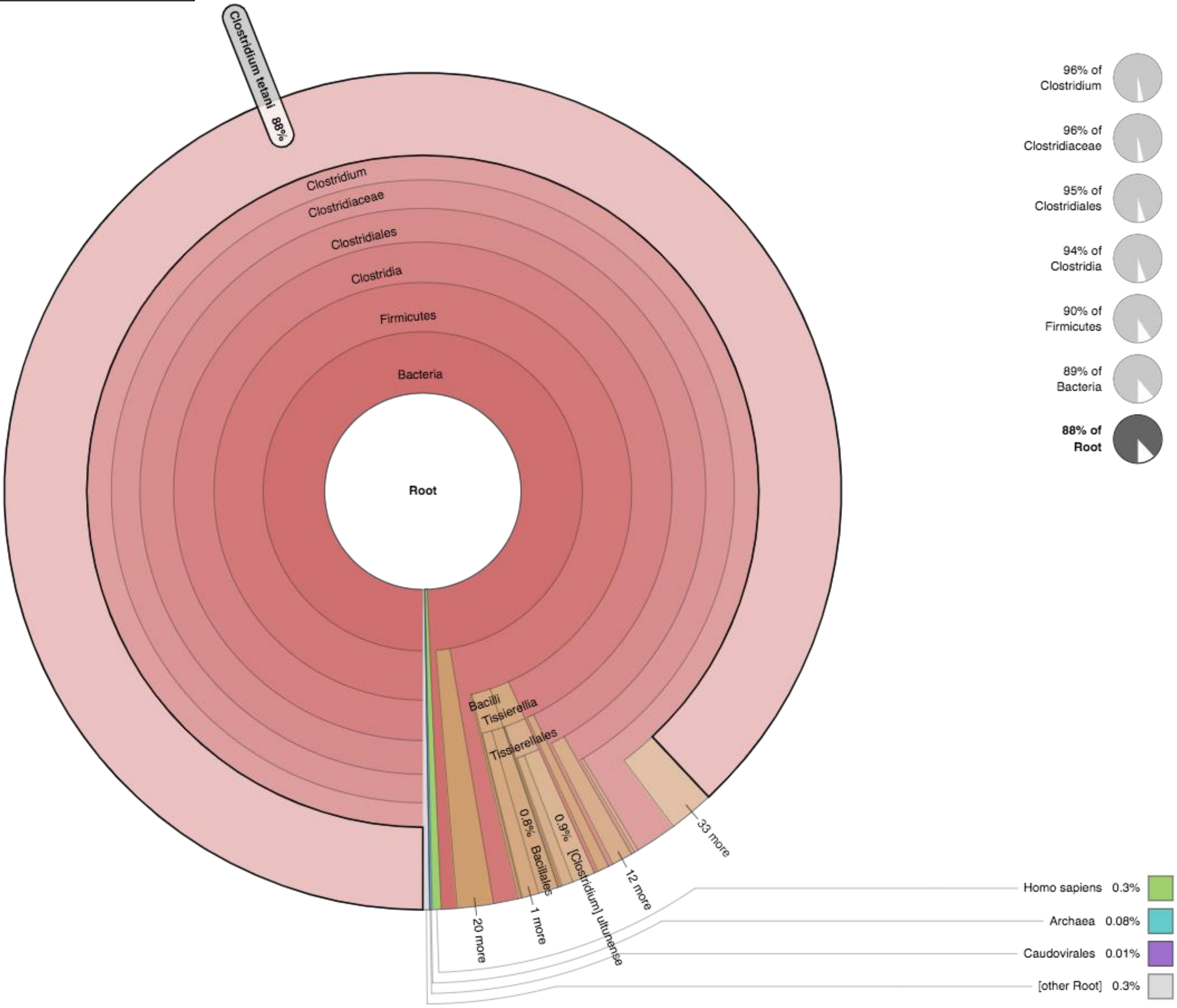

Supplementary figure 2: generated whole genome Circular Viewer on the Bacterial and Viral Bioinformatics Resource Center (BV-BRC).

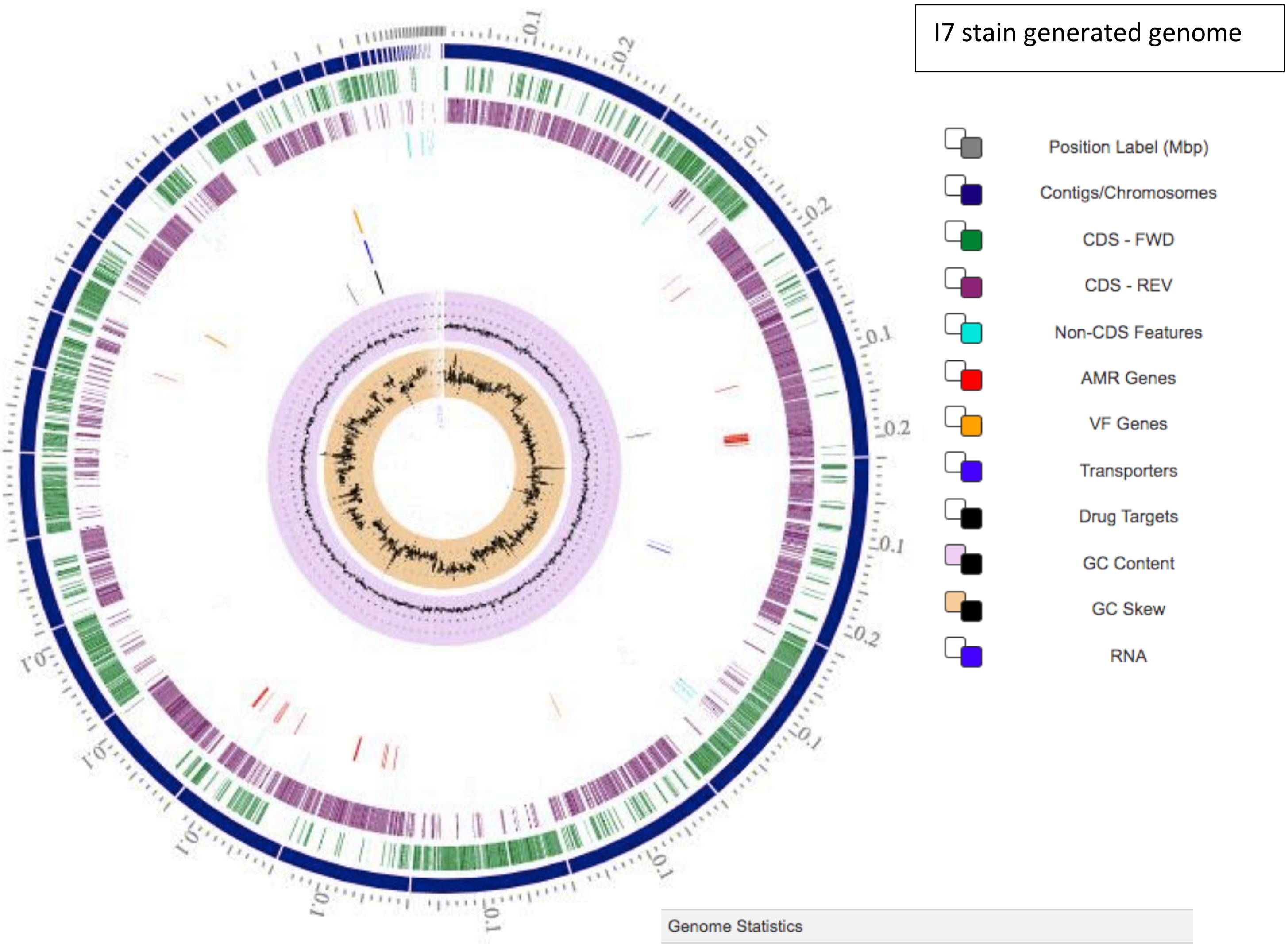

Genome Statistics	
**Contigs**	51
**Genome Length**	2880792
**GC Content**	28.504244
**Contig L50**	7
**Contig N50**	181715
Annotation Statistics	
**tRNA**	54
**rRNA**	7
**CDS**	2904
**CDS Ratio**	1.0080562
**Hypothetical CDS**	734
**Hypothetical CDS Ratio**	0.35640496
**PLFAM CDS**	2793
**PLFAM CDS Ratio**	0.96177685
Genome Quality	
**Coarse Consistency**	99.5
**Fine Consistency**	99
**CheckM Completeness**	99.8
**CheckM Contamination**	0.2
**Genome Quality**	Good
Isolate Info	

I44 stain generated genome

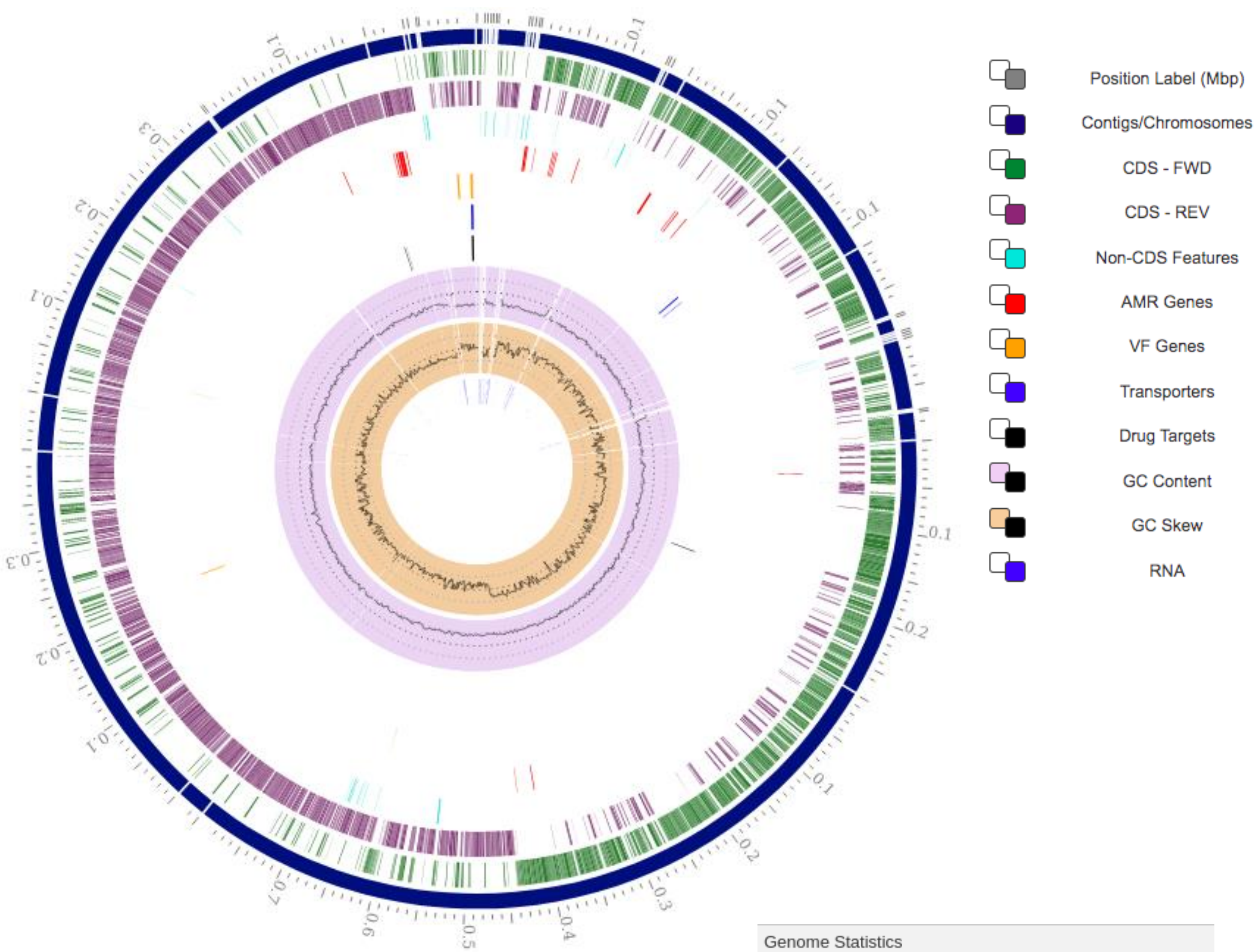

Genome Statistics	
**Contigs**	39
**Genome Length**	2858093
**GC Content**	28.68655
**Contig L50**	3
**Contig N50**	349193
Annotation Statistics	
**tRNA**	52
**rRNA**	14
**CDS**	2841
**CDS Ratio**	0.99401945
**Hypothetical CDS**	685
**Hypothetical CDS Ratio**	0.34494895
**PLFAM CDS**	2796
**PLFAM CDS Ratio**	0.9841605
Genome Quality	
**Coarse Consistency**	99.5
**Fine Consistency**	99
**CheckM Completeness**	100
**CheckM Contamination**	0.3
**Genome Quality**	Good

Supplementary figure 3: genomic comparisons using coding DNA sequences (CDS) by maps with multiple BLAST comparisons using Proksee online (https://proksee.ca/) (Grant et al., 2023), including height published *C. tetani* strains genomes : E88 (GCA_000007625.1) like reference genome, Havard (GCF_004119355.1), NIID-071400-001 (GCF_033128285.1), KHSU-254310-026 (GCF_033128265.1), KHSU-144316-041 (GCF_033128185.1), ATCC 453 (GCF_000762325.1), Mfbjulcb2 (GCF_003013635) and the two ancients *C. tetani* strains Q7452 (GCF_949357665.1) and Q7451 (GCF_949357675.1). (A): whole genome comparison, (B) plasmid comparison, (C1-3) different very heterogeneous genetic islands between all strains showing genes absence in comparison with reference genome C. tetani E88.

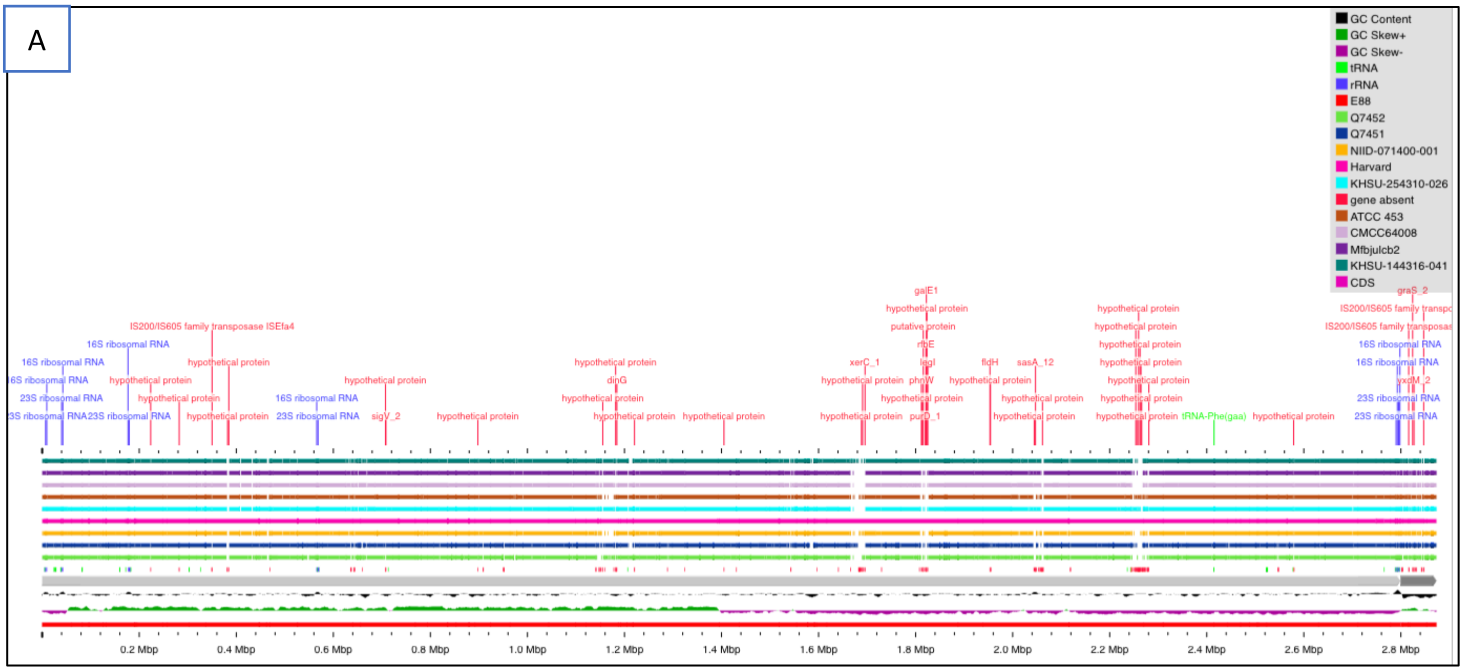

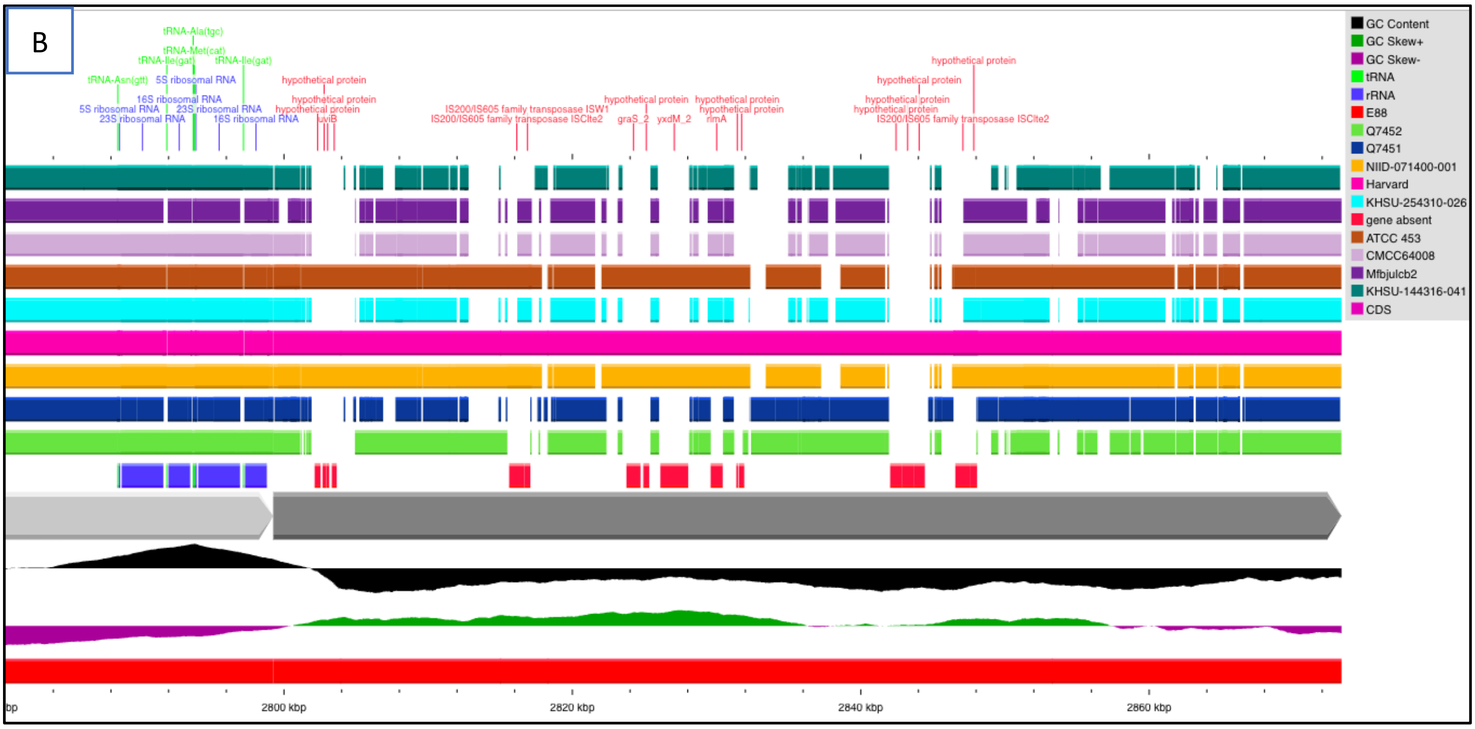

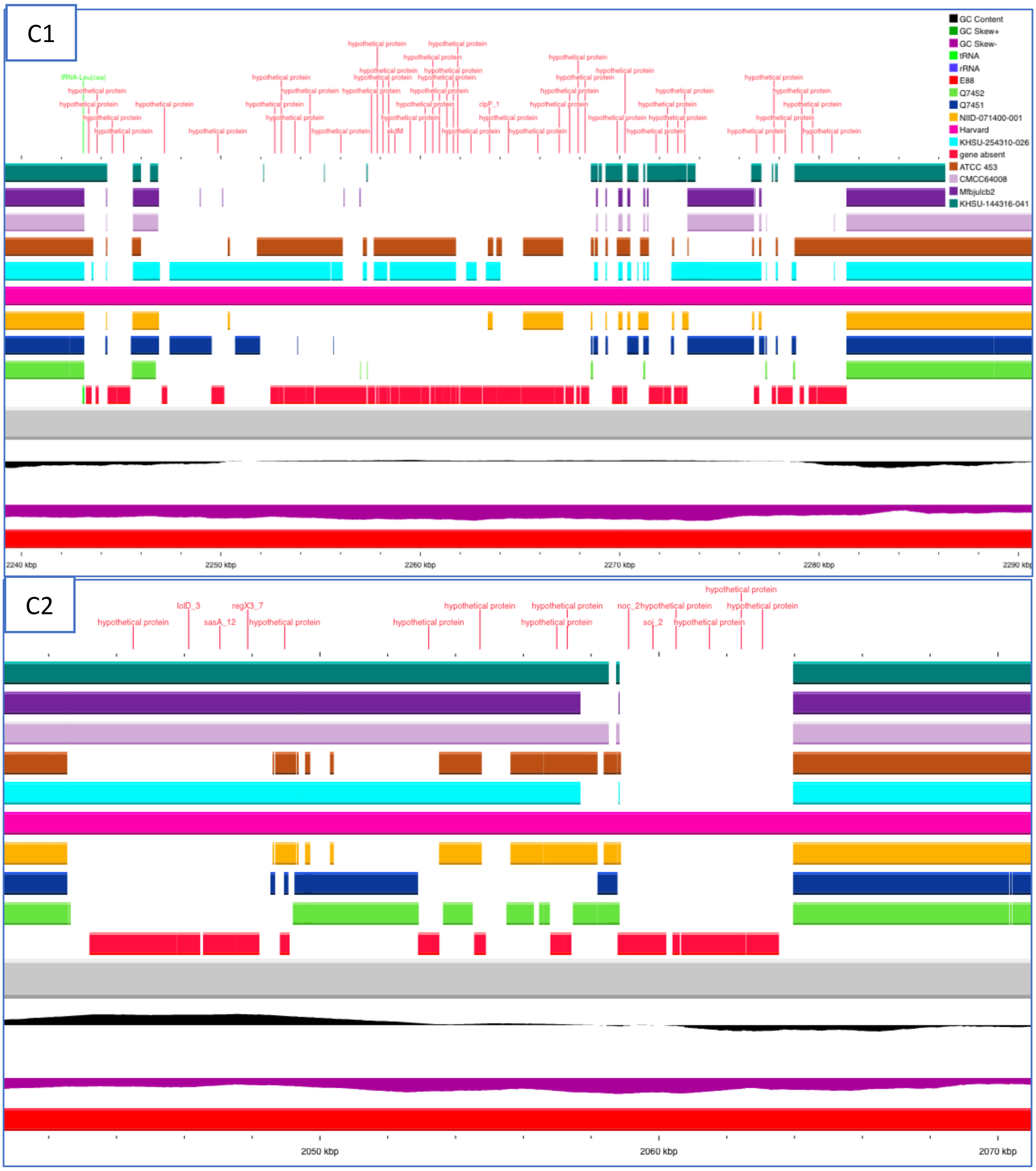
C1
GC Content
GC Skew+
GC Skew-
tRNA
rRNA
E88
Q7452
Q7451
NIID-071400-001
Harvard
KHSU-254310-026
gene absent
ATCC 453
CMCC64008
Mfbjulcb2
KHSU-144316-041
tRNA-Leu(caa)
clpP_1
xkdM
2240 kbp
2250 kbp
2260 kbp
2270 kbp
2280 kbp
C2
hypothetical protein
lolD_3
sasA_12
regX3_7
noc_2
soj_2
2050 kbp
2060 kbp
2070 kbp

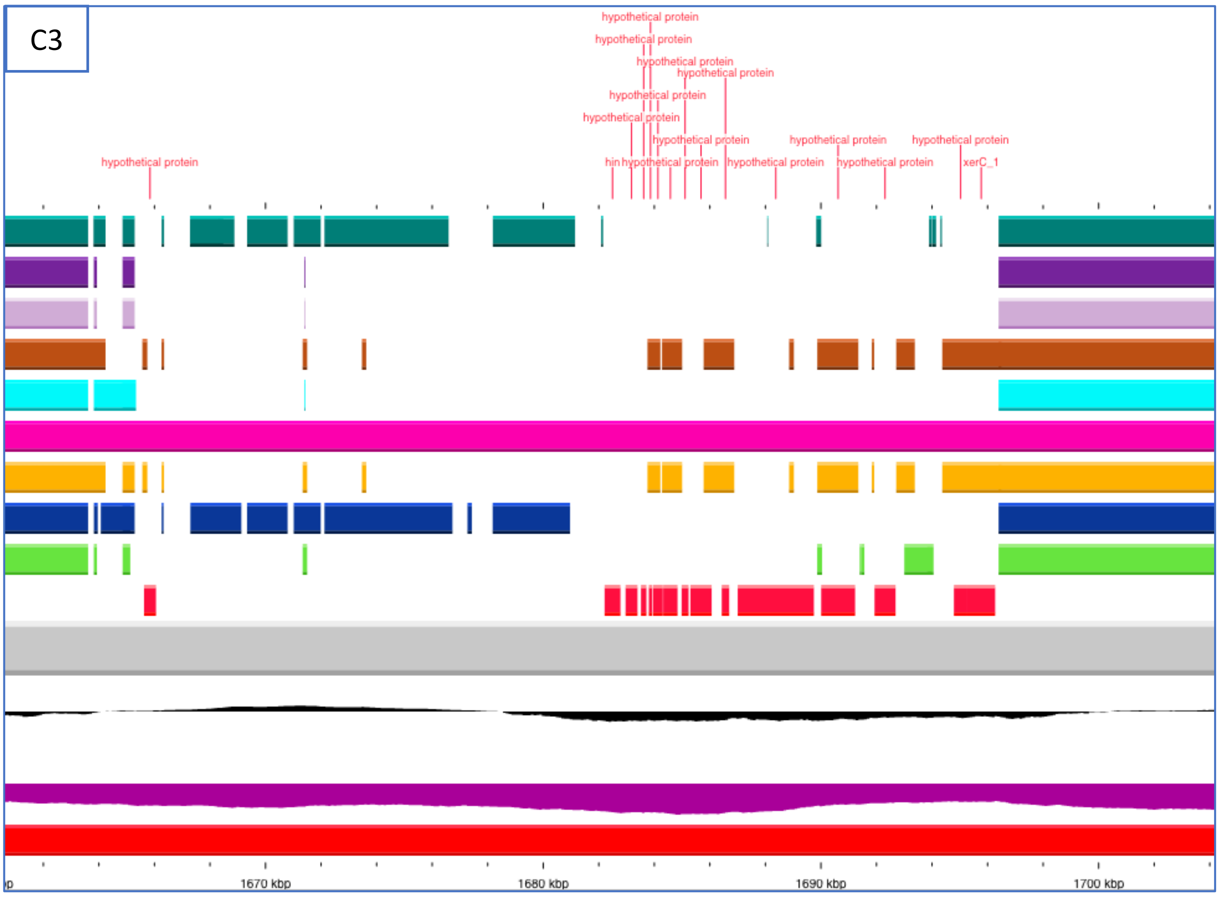

C3
hypothetical protein
hypothetical protein
hypothetical protein
hypothetical protein
hypothetical protein
hypothetical protein
hypothetical protein
hypothetical protein
hypothetical protein
hypothetical protein
hin
hypothetical protein
hypothetical protein
hypothetical protein
xerC_1
1670 kbp
1680 kbp
1690 kbp
1700 kbp
